# Preparation of two kinds of immunocastration vaccines and their immune effects on male goats

**DOI:** 10.5713/ab.24.0811

**Published:** 2025-04-11

**Authors:** Fuqiang Pan, Yumeng Guo, Panpan Cheng, Wei Qian, Mengdi Han, Qing Yi, Huihui Xie, Meng Cao, Yanqiuhong Li, Yuke Jia, Jiankun Cui, Xinbao Gong, Ziye Zhu, Fugui Fang, Yinghui Ling, Yunsheng Li, Jian Li, Ya Liu

**Affiliations:** 1 College of Veterinary Medicine, Anhui Agricultural University, Hefei, China; 2 Biological and Food Engineering College, Fuyang Normal University, Fuyang, China; 3 Anhui Divinity Biological Products Co., Ltd., Bozhou, China; 4 College of Veterinary Medicine, Nanjing Agricultural University, Nanjing, China; 5 College of Animal Science and Technology, Anhui Agricultural University, Hefei, China

**Keywords:** Goat, Gonadotropin-releasing Hormone (GnRH), Immunocastration, Luteinizing Hormone Receptor (LHR), Vaccine

## Abstract

**Objective:**

Post-pubertal male goats exhibit undesirable behaviors (fighting, mounting) and reduced growth due to high testosterone, while traditional castration causes stress. Immunocastration offers a humane alternative. This study aimed to develop effective immunocastration vaccines.

**Methods:**

The gonadotropin-releasing hormone (GnRH) octamer vaccine (G8), luteinizing hormone receptor (LHR) and GnRH octamer tandem vaccine (LG) were developed and synthesized for this study. Forty 3-month-old male goats were randomly divided into four groups, and subjected to different treatments: surgical castration (SC group), immunization with the G8 vaccine (G8 group), immunization with the LG vaccine (LG group), or left intact (non-castration [NC] group). After the first immunization, serum antibodies and testosterone levels, as well as body weight, body size, and scrotal size, were measured at various time points. Testicular size and slaughter rate were measured at the time of slaughter, 20 weeks after the first immunization.

**Results:**

Both vaccines effectively elicited the corresponding antibodies in male goats; the testosterone levels in the G8 and LG groups were significantly reduced compared to the NC group (p<0.01). Four weeks after the first immunization, this trend persisted throughout the experiment; the testicular organ index and size of the G8 group were significantly (p<0.05) smaller than those of NC group at slaughter. In comparison to the NC group, the seminiferous tubule diameter in the G8 and LG groups was significantly reduced (p<0.01), accompanied by a notable decrease in Leydig Cells and various stages of spermatogenic cells. Additionally, the weight gain of goats in the SC group was significantly lower than that of other groups two weeks after the first immunization (p<0.05).

**Conclusion:**

The two immunocastration vaccines developed in this study effectively inhibit the testicular development and spermatogenesis in male goats, leading to a reduction in testosterone levels.

## INTRODUCTION

After sexual maturity, male goats will exhibit fighting and mounting behaviors due to the increase of testosterone level in the blood [1,2], resulting in decreased feed intake, increased energy consumption and slower growth rate, and the increase of testosterone level will increase the content of branched-chain fatty acids in fat, resulting in mutton odor [3].

The development and maintenance of male animal gonads, as well as the secretion of testosterone, are mainly regulated by the hypothalamic pituitary testis axis (HPTA). Gonadotropin-releasing hormone (GnRH) can stimulate the synthesis and release of luteinizing hormone (LH) and follicle-stimulating hormone (FSH) [4], and in turn regulate the secretion of related gonadal steroid hormones and gamete formation [5]. FSH indirectly affects sperm production by regulating the function of supporting cells [6]. LH can promote the synthesis and release of testosterone in Leydig Cells [7]. Therefore, the secretion of testosterone is directly regulated by LH and when testosterone levels reach a certain concentration, it will prompt male animals to exhibit the above-mentioned behaviors.

In practical production, male livestock are usually castrated to reduce their aggressiveness [8] and improve the meat quality of livestocks [9]. This is the most direct method to eliminate testosterone secretion. However, traditional castration methods not only cause significant stress [10], but also pose a risk of postoperative infection and death [11]. With the development of castration-related technologies and attention to animal welfare, mechanical castration methods are now discouraged, and castration without analgesia or anesthesia will be banned in many countries [12]. Therefore, it is necessary to develop a welfare-friendly and gentler approach to replace mechanical castration. Immunocastration can effectively reduce the stress response and, to some extent, improve the growth rate of male animals [13]. Therefore, it can be used as an alternative to mechanical castration.

During past decades, some studies have been conducted on goat immunocastration. Lents et al [14] immunized 8-month-old male goats with Bopriva^TM^ and found that the size of the scrotum and testicles, sperm motility, and quantity in the immunized group were significantly reduced. Wassie et al [15] immunized goats with Kisspeptin-54 DNA vaccine, and found that the goats produced high levels of antibodies against kisspeptin and exhibited reduced serum testosterone. Ding et al [16] found when male goats were immunized with a recombinant B2L and KISS1 DNA vaccine, the serum FSH and LH levels were decreased in goats, the scrotal circumference was reduced, and the libido was diminished. However, there are not as many studies on immunocastration in goats as in other animals [17,18], and immunocastration vaccines for goats are still in the experimental stage, which have some defects to varying degrees. As one of the main meat animals, developing a vaccine that can effectively immunize goats against castration is of great value and significance.

Based on relevant experiments in recent years, there are many potential target sites in the research on immunocastration vaccines, among which GnRH is the most studied. However, owing to the small molecular weight of a single GnRH molecule, its immunogenicity is poor. By serializing or polymerizing multiple GnRH monomers or connecting to immune-enhancing proteins, the immunogenicity of GnRH can be effectively improved, thereby enhancing the immune efficacy of vaccines [15,19]. At the same time, the development of the testes and the generation of sexual behavior in male mammals are regulated by multiple hormones in HPTA. If a multivalent vaccine targeting multiple hormones or their receptors that stimulate testicular development can be developed, better immunocastration effects may be achieved. Here, we constructed an octamer GnRH8 composed of GnRH and its variants and connected it with antigenic epitopes of LH receptor (LHR) to form the LHR-GnRH8 (LG) antigen. Then, GnRH8 and LG were fused and expressed with the diphtheria toxin T cell helper sequence (DTT), respectively, and validated in male goats.

## MATERIALS AND METHODS

### Animal care

All procedures involving goats were approved by the Animal Care and Use Committee of the Anhui Agricultural University (No. AHAU20208025).

### Experimental animals

The study was conducted at Linquan Tian yuan Animal Husbandry Co., Ltd. (Linquan, Anhui, China). Forty healthy 3-month-old male Wanlin White goats of similar weights (16±2.5 kg) were raised in the same pen. They were provided ad libitum with water and natural light and fed three times a day with the same forage.

### Antigen design and plasmid construction

The GnRH sequence and its seven analogs were connected using Gly-Gly-Ser sequences, resulting in the construction of a GnRH8 (Supplement 1A). The GnRH8 sequence was combined with the previously designed LHR antigenic epitope peptide sequence [20] to construct the LG sequence (Supplement 1B). GnRH8 and LG sequences were separately inserted into existing pET28a-DTT plasmids in the laboratory [20], resulting in the construction of pET28a-GnRH8-DTT and pET28a-LHR-GnRH8-DTT plasmids (Used for preparing G8 and LG immunocastration vaccines) (Supplements 2A, 2B).

The DNA sequence encoding GnRH8 was inserted into the pMAL-C2X vector to construct the pMAL-C2X-GnRH8 recombinant plasmid (used for preparing coated protein in Elisa for GnRH antibody detection). The pGEX-6P-1-LHR recombinant plasmid (used for preparing coated protein in Elisa for LHR antibody detection) was previously designed and preserved in our laboratory (Supplements 2C, 2D).

### Expression and purification of proteins

The aforementioned plasmids were transferred into *Escherichia coli* strain BL21 (DE3), and expression of the target proteins was induced with IPTG (0.1 mM). Proteins were purified according to the corresponding instructions provided in the kits (PurKine Maltose binding protein labeled dextrin resin BMR2020; Abbkine, Wuhan, China; BeyoGold His tag Purification Resin [denaturation resistant formulation], P2233-10 mL; Beyotime, Jiangsu, China) (Supplement 3). The coated proteins (maltose binding protein [MBP]-GnRH8 and glutathione S-transferase [GST]-LHR) were concentrated using ultrafiltration at 4°C. The vaccine proteins (GnRH8-DTT and LHR-GnRH8-DTT) were low temperature freeze-dried and preservation at −80°C.

### Vaccine preparation

The freeze-dried protein (GnRH8-DTT and LHR-GnRH8-DTT) was dissolved in physiological saline and white oil adjuvant (MONTANIDETM ISA201 VG, U51021, SEPPIC, France) in a 1:1 volume ratio and emulsified thoroughly. Each milliliter of the vaccine contained 1 mg of protein.

### Experimental design

The experiment was performed using a completely randomized design. The 40 goats were randomly allocated to four groups (n = 10). The surgical castration (SC) group underwent surgical removal of testes at three months old. The non-castration (NC), GnRH8-DTT immunocastration (G8), and LHR-GnRH8-DTT immunocastration (LG) groups were subcutaneously injected with 1 mL of physiological saline and adjuvant, G8 vaccine, and LG vaccine, respectively, at three months old. Four weeks later, the same dose and immune pathways were used to strengthen the immunity.

At 0, 2, 4, 8, 12, 16, and 20 weeks after the first immunization, blood was collected from each goat through the jugular vein to measure the serum testosterone and antibodies levels. The body weight, body size, scrotal transverse diameter, longitudinal diameter, and circumference were measured. Twenty weeks after the first immunization, all goats were slaughtered, and the slaughter rate, muscle pH, weight, and testes size (the average values of bilateral testes) were measured, and testicular tissue structure was observed.

### Determination of slaughter rate and meat pH

Prior to slaughter, the goats were fasted for 24 h and deprived of water for 12 h before weighing. Weight was recorded as fasting live weight. After slaughter, the goats were skinned, and their heads, hooves, and internal organs were removed (retaining the kidneys and renal fat). The weight obtained after hanging upside down and resting for 30 min was recorded as carcass weight.


Slaughtering rate (%)=carcass weight/fasting live weight×100%

A portable meat puncture pH meter (PH-K21; Beijing Brad Technology Development Co., Ltd., Beijing, China) was used to test muscle pH at a distance of 10 cm from the knee joint on the left hind limb of the carcass.

### Serum antibody level detection

The purified fusion proteins MBP-GnRH8 and GST-LHR were diluted with phosphate-buffered saline (PBS) to a concentration of 2 μg/mL. Then, 96-well plates were coated with diluted MBP-GnRH8 or GST-LHR, and the corresponding antibody levels were detected according to a previously described before [20] . Briefly, 100 μL of coated protein solution was added to each well of the plates and incubated at 4°C overnight. The plates were then washed with Phosphate-Buffered Saline with Tween-20 (PBST) and sealed with sealing solution. The diluted serum (Dilute 100x) was added to the wells of the coated 96-well plate and incubated at 37°C for 40 min. After washing, the plates were incubated with diluted (1:50,000) rabbit anti-goat IgG Horseradish Peroxidase (HRP) antibody (A21030; Abbkine, Wuhan, China),100 μL/well, at 37°C for 40 min. The TMB chromogenic solution was added to the plates and incubated at 37°C for 15 min in the dark. The reaction was terminated and the optical density was determined.

### Testosterone detection

Testosterone (T) levels were quantified using a chemiluminescence immunoassay (CLIA) (MINDRAY, Shenzhen, China). Briefly, samples were initially mixed with testosterone antibodies labeled with alkaline phosphatase and magnetic latex reagents. Subsequently, a chemiluminescent substrate, 3-[2-spiroadamantane]-4-methoxy-4-[3-phosphoryloxy]-Phenyl-1,2-dioxetane, was introduced to generate chemiluminescent signals that were quantified by counting the emitted photons. The minimum detection limit is 0.1 ng/mL, with a detection range from 0.1 ng/mL to 16.0 ng/mL, and coefficient of variation (CV) values within the range of 5.82% for intra-assay and 4.22% for inter-assay, respectively. The correlation coefficient of the standard curve is 0.9900.

### Histomorphological observation of testis

As described previously [18], the testes were collected and fixed in 4% paraformaldehyde, dehydrated in ethanol, embedded in paraffin wax, sectioned, and stained with hematoxylin and eosin (HE) staining [21]. The tissue structure was observed under microscope (BX51). Images were taken (Nikon Digital Sight DS-SMC camera; Nikon, Tokyo, Japan) and saved.

We randomly selected 10 morphologically intact, circular, or elliptical seminiferous tubules from the HE slices of each sample and measured the longest diameter (μm) in the seminiferous tubule section using ImageJ software for recording and subsequent data analysis.

### Statistical analysis

All statistical analyses were performed using IBM SPSS Statistics 24 software, and significance analysis was performed using one-way ANOVA between multiple groups, followed by Tukey post hoc test; the results are shown as the mean±standard error of the mean, with p*<*0.05 indicating significant differences, p<0.01, indicating extremely significant difference, and p*>*0.05, indicating no significant differences. The results were plotted using the GraphPad Prism software 3.

## RESULT

### Serum antibody levels

**Figure 1:**
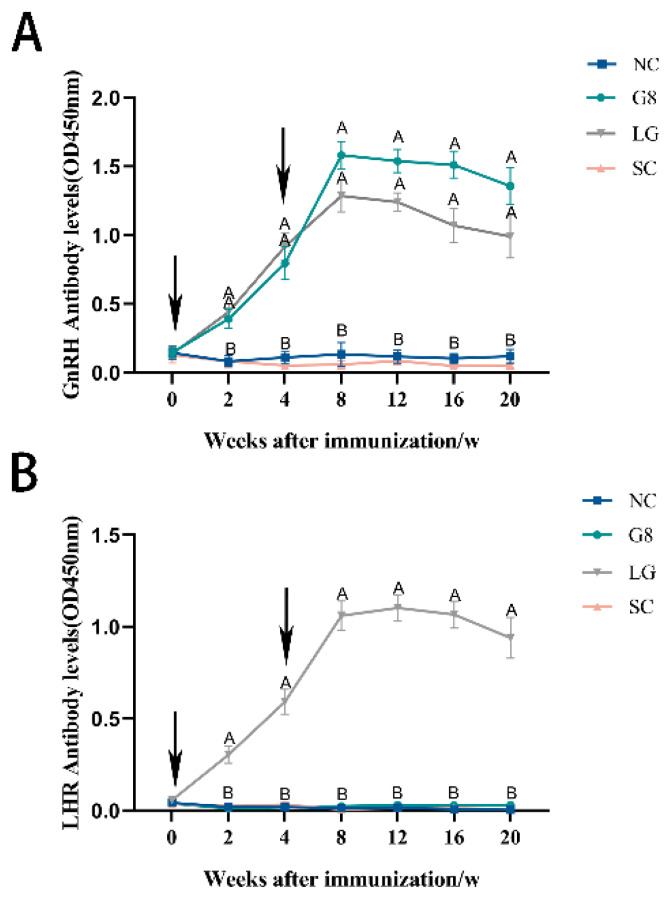
Changes in serum antibody levels. A. Elisa analysis of anti-GnRH antibody levels. B. Elisa analysis of anti-LHR antibody levels. Arrows indicate vaccination time. ^A,B^ Different uppercase letters represent p<0.01. NC, non-castration; G8, GnRH8-DTT immunocastration; LG, LHR-GnRH8-DTT immunocastration; SC, surgical castration; GnRH8, GnRH octamer; GnRH, gonadotropin-releasing hormone; DTT, diphtheria toxin T cell helper sequence; LHR, luteinizing hormone receptor.

**Figure 2:**
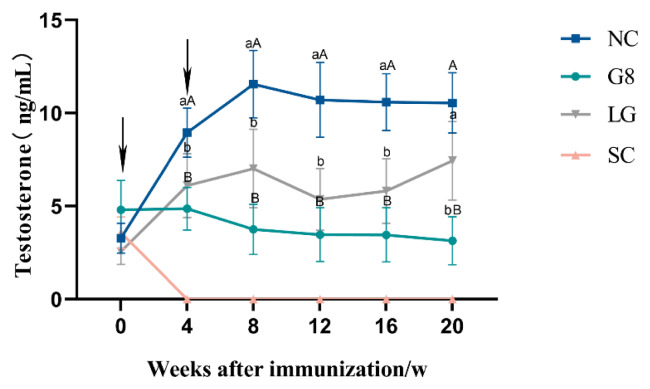
Changes in serum testosterone levels at different stages after the first immunization. Arrows indicate vaccination times. ^a,b^ Different lowercase letters represent p<0.05; ^A,B^ different uppercase letters represent p<0.01. NC, non-castration; G8, GnRH8-DTT immunocastration; LG, LHR-GnRH8-DTT immunocastration; SC, surgical castration; GnRH8, GnRH octamer; GnRH, gonadotropin-releasing hormone; DTT, diphtheria toxin T cell helper sequence; LHR, luteinizing hormone receptor.

As shown in Figures 1, 2 weeks after the first immunization, both the G8 and LG groups of goats began to produce antibodies, which were significantly higher than those of the NC and SC groups (p<0.01), and continued until the end of the experiment. At 8 weeks, GnRH antibody levels in the G8 and LG groups peaked. At 12 weeks, the LHR antibody levels in the LG group peaked. Both antibodies gradually and slowly decreased after reaching their peak but maintained a relatively high level until the end of the experiment.

### Serum testosterone levels

As shown in Figure 2, testosterone levels in the SC group remained undetectable four weeks after the initial immunization until 20 weeks. From 4 to 16 weeks, the serum testosterone levels in the NC group were significantly higher than those in the G8 and SC groups (p*<*0.01), and significantly higher than those in the LG group (p*<*0.05). Serum testosterone levels in the G8 group were generally lower than those in the LG group and maintained a steady downward trend.

### The effect of immunocastration on the development of testicles and scrotum in goats

#### The impact on testicular organ index

**Figure 3:**
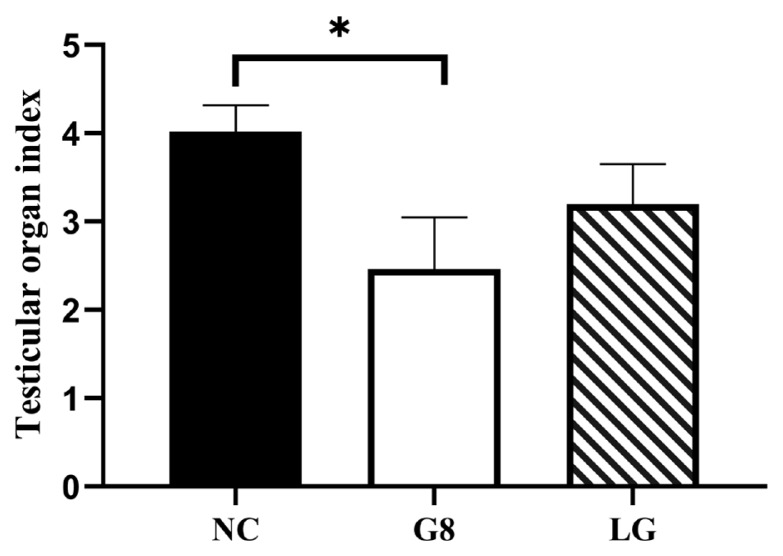
Effect of vaccine on testicular organ index. Testicular organ index = average weight of left and right testicles (g)/carcass weight (kg)×100%.* p<0.05. NC, non-castration; G8, GnRH8-DTT immunocastration; LG, LHR-GnRH8-DTT immunocastration; SC, surgical castration; GnRH8, GnRH octamer; GnRH, gonadotropin-releasing hormone; DTT, diphtheria toxin T cell helper sequence; LHR, luteinizing hormone receptor.

As shown in Figure 3, Twenty weeks after the first immunization, the testicular organ index of the G8 group was significantly lower than that of the NC group (p*<*0.05).

#### The impact on testicular size

**Figure 4:**
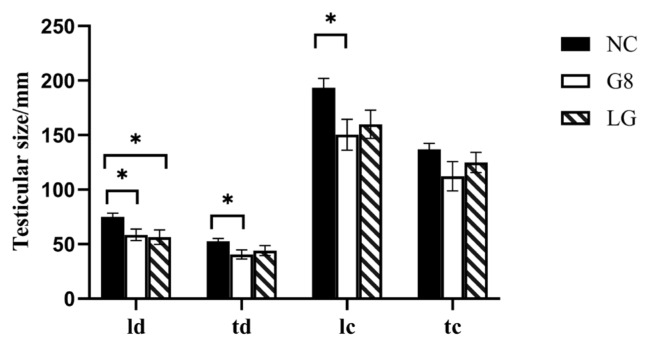
Effect of vaccines on testicular size. All statistical values represent the average values of the left and right testicles of the same goat. * p<0.05. NC, non-castration; G8, GnRH8-DTT immunocastration; LG, LHR-GnRH8-DTT immunocastration; SC, surgical castration; GnRH8, GnRH octamer; GnRH, gonadotropin-releasing hormone; DTT, diphtheria toxin T cell helper sequence; LHR, luteinizing hormone receptor; ld, longitudinal diameter of testes; td, transverse diameter of testes; lc, longitudinal circumference of testes; tc, transverse circumference of testes.

As shown in Figure 4, Twenty weeks after the first immunization, the transverse diameter (td), longitudinal diameter (ld), and longitudinal circumference (lc) of the testes in the G8 group were significantly smaller than those in the NC group (p<0.05), while the longitudinal diameter of the testes in the LG group was significantly smaller than that in the NC group (p*<*0.05).

#### Effects on the development of testes

**Figure 5:**
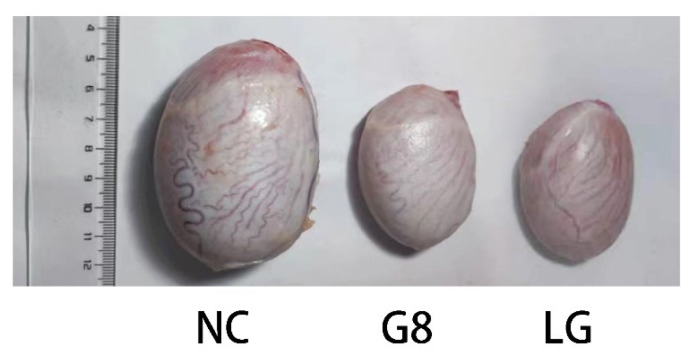
Picture of testicles. The left testicle was from the NC group, the middle testicle was from the G8 group, and the right testicle was from the LG group. NC, non-castration; G8, GnRH8-DTT immunocastration; LG, LHR-GnRH8-DTT immunocastration; GnRH8, GnRH octamer; GnRH, gonadotropin-releasing hormone; DTT, diphtheria toxin T cell helper sequence; LHR, luteinizing hormone receptor.

Twenty weeks after the first immunization, the testes of all goats were collected and the development of testes was evaluated. As shown in Figure 5, the testes of the immunocastrated goats were remarkably smaller than those of the NC group.

**Figure 6:**
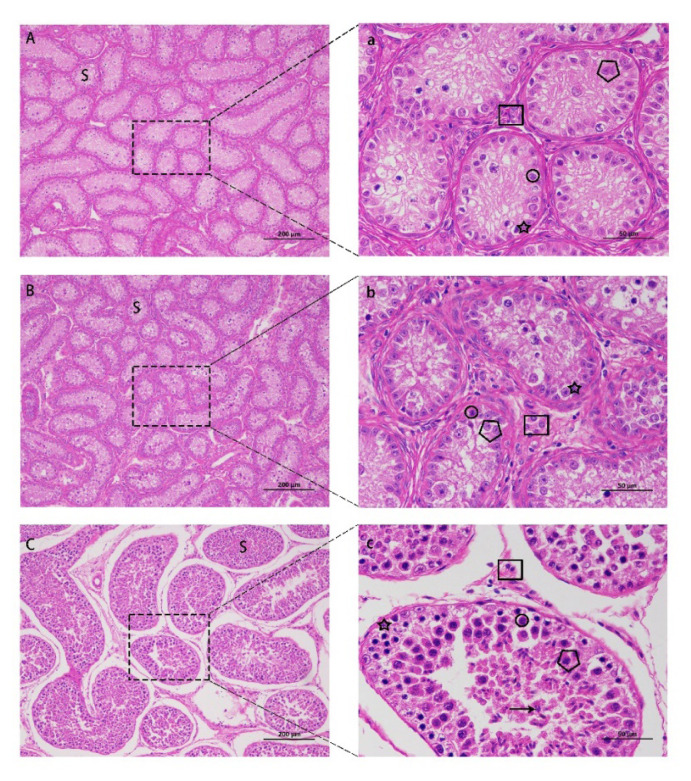
Histological observations of the testes. A. G8 group (100×); B. LG group (100×); C. NC group (100×); a. G8 group (100×); b. LG group (100×); c. NC group (100×); S, seminiferous tubules; solid box, Leydig cells; circle, spermatogonia; pentagram, supporting cells; pentagon, primary spermatocytes; arrow, sperm. The scale of A, B, C is 200 μM; The scale of a, b, c is 50 μM. G8, GnRH8-DTT immunocastration; LG, LHR-GnRH8-DTT immunocastration; NC, non-castration; GnRH8, GnRH octamer; GnRH, gonadotropin-releasing hormone; DTT, diphtheria toxin T cell helper sequence; LHR, luteinizing hormone receptor.

HE staining was performed on the testicular sections, and the results are shown in Figure 6. The development of spermatogenic tubules in the NC group was normal, with a loose and regular arrangement, and testicular interstitial cells were evenly distributed between the tubules (Figure 6C). Sertoli cells, spermatogonia, and primary spermatocytes developed normally and were densely packed, with a large number of spermatids or sperm present in the lumen (Figure 6C). The seminiferous tubules of the testes in the G8 and LG groups were not fully developed and were significantly atrophied compared to those in the NC group. The arrangement between the tubules was very tight, and the interstitial cells of the testes were significantly reduced (Figures 6A, 6B). The seminiferous tubules were filled with filamentous stroma, and the development of testicular-supporting cells, spermatogonia, and primary spermatocytes was insufficient and significantly reduced in number, with no sperm formation in the lumen (Figures 6A, 6B).

**Figure 7:**
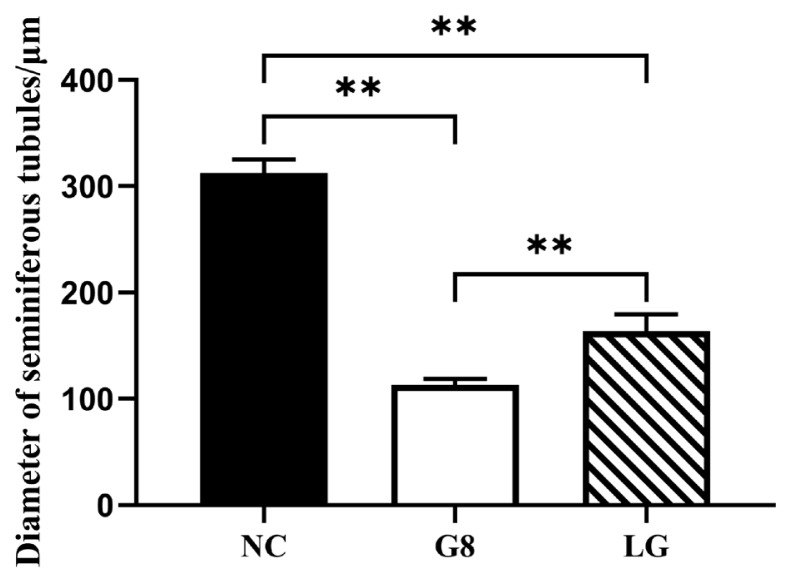
Diameter of seminiferous tubules. Ten seminiferous tubules with complete morphology and circular cross-section from each HE slice were randomly selected for diameter measurement, and the longest diameter in the entire circle was uniformly recorded for relevant statistical analysis. ** p<0.01. NC, non-castration; G8, GnRH8-DTT immunocastration; LG, LHR-GnRH8-DTT immunocastration; GnRH8, GnRH octamer; GnRH, gonadotropin-releasing hormone; DTT, diphtheria toxin T cell helper sequence; LHR, luteinizing hormone receptor.

We measured the average diameter of seminiferous tubules in the three groups of testicular HE sections. As shown in Figure 7, the average diameter of the seminiferous tubules in the HE section of the G8 group was significantly smaller than that in the LG and NC groups (p*<*0.01), and the average diameter of the seminiferous tubules in the LG group was significantly smaller than that in the NC group (p<0.01).

#### Effects on scrotal development

As shown in Table 1, immunocastration significantly inhibited the growth of the goat scrotum, and G8 immunity had a better inhibitory effect on the scrotum than LG immunity.

### Effects on the productive performance of goats

#### The impact on weight gain

As shown in Table 2, during the first week after castration, the goats in the SC group experienced a decrease in average body weight owing to intense stress. In contrast, the immunocastration and intact groups showed some weight gain, with net weight gain (NWG) significantly higher than that of the SC group. Two weeks after the initial vaccination, the average NWG in the G8 group was significantly higher than that in the SC group. There was no significant difference in NWG among the groups of goats for the remaining duration of the experiment.

#### The impact on body size

As shown in Table 3, there was no significant difference in initial body size among the groups. At the final slaughter, compared with the NC group, LG immunization remarkably promoted an increase in body height (p*<*0.05). The method of castration and whether castration was performed had no significant effect on the body length and tube circumference of the male goats.

#### Effects on slaught rate and muscle pH

**Figure 8:**
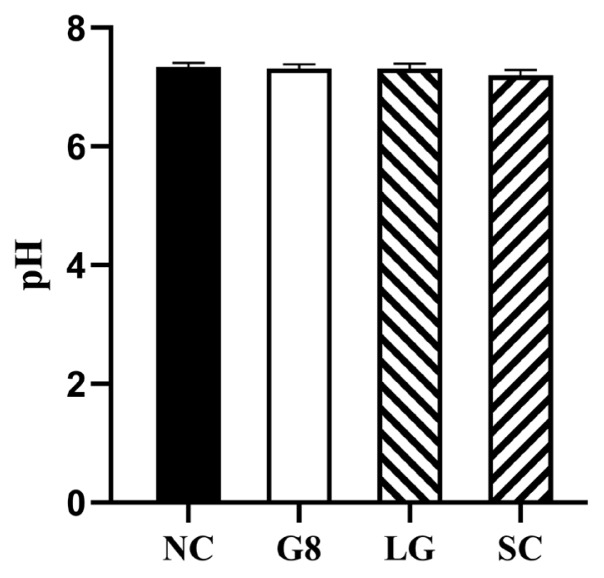
Muscle pH of Goats. All goats were measured at the 30th minute after slaughter, using a meat pH tester at the same location on the thigh of the left hind limb of all goats, and waiting until the readings stabilized to record them. NC, non-castration; G8, GnRH8-DTT immunocastration; LG, LHR-GnRH8-DTT immunocastration; SC, surgical castration; GnRH8, GnRH octamer; GnRH, gonadotropin-releasing hormone; DTT, diphtheria toxin T cell helper sequence; LHR, luteinizing hormone receptor.

Twenty weeks after the first immunization, the method of castration and whether castration was performed had no significant effect on the slaughter rate and muscle pH of goats (p>0.05) (Table 4; Figure 8).

## DISCUSSION

After the first immunization, the G8 and LG groups began to produce antibodies in the 2nd week. Around weeks 8th to 12th, the antibody levels in the G8 and LG groups peaked. This is consistent with the results of previous experiments [18,22]. After modification, G8 and LG in this experiment can be used as an immunogen to stimulate the body to produce corresponding antibodies quickly, reaching a higher level within a short period.

The elevated levels of antibodies in the G8 and LG groups disrupted the hormonal equilibrium in the HPTA, resulting in a gradual decrease in testosterone levels rather than a significant increase, as observed in the NC group, and remained low until the conclusion of the experiment. This is similar to most of the experimental results [23,24]. There is mutual regulation and influence between testosterone and the testes [25]; testosterone can affect the development of the testes, and the testes are the main organs that secrete testosterone. Leydig Cells in the testes are the main cells that secrete testosterone [26], and Leydig Cells are mainly regulated by LH [27]. Thus, both the G8 and LG groups ultimately led to a significant decrease in the number of Leydig Cells, a significant decrease in sperm production related cells, and testosterone secretion [28], which resulted in a significant reduction in the transverse and longitudinal diameters of the testes in both groups compared to the NC group, and thus a significant reduction in the scrotum in both groups compared to the NC group. The results of numerous experiments [29,30] were also consistent with this experiment. Furthermore, because the serum testosterone levels showed a result as NC group>LG group> G8 group, the size and organ index of the testes during slaughter also showed a trend of NC group>LG group>G8 group. However, the significantly lower testosterone levels in the G8 group than those in the LG group were unexpected. We speculate that the likely reason for this is that the amount of LHR and GnRH targeted proteins alone in the LG group was lower than that of GnRH in the G8 group at the same immunization dose; therefore, the level of antibodies induced was not as high as that in the G8 group. It is also possible that more antigens were immunised in the LG group than in the G8 group, which dispersed the body’s ability to produce antibodies, resulting in lower antibody levels than in the G8 group. This is illustrated by the fact that the level of GnRH antibodies in the LG group in Figure 1 is lower than that in the G8 group, and presumably, the level of antibodies against LHR is also not sufficient to completely block the biological function of LHR. Thus, resulting HPTA can still achieve a certain low level of balance. This explains why the inhibitory effect on testosterone secretion was lower in the LG group than that in the G8 group. However, this is precisely because the LG group did not show a significant decrease in testosterone levels compared to the G8 group, which led to a faster weight gain rate in the LG group than in the G8 and SC groups during the late stage of immunization.

The castration of rams can effectively reduce the occurrence of climbing and fighting behaviors while reducing the mutton smell [31] and improving growth efficiency. However, testosterone not only regulates sexual behavior but also plays an important role in protein synthesis, body size, and bone mass maintenance in the body [32,33]. Animals after surgical castration often rapidly become obese due to the loss of testosterone secretion [34], and the slaughter rate and lean meat rate actually decrease compared to before castration [35]. Within 0 to 2 weeks after the start of the experiment, the weight gain of the SC group significantly decreased and even showed negative growth. This may be due to the decrease in food intake and significant weight loss caused by stress, such as trauma, pain, or wound infection, in the SC group after surgical castration. Many studies [11,36] have pointed out that surgical castration can cause strong stress on animals and affect their production performance, confirming the results of this experiment. In contrast, the G8 and LG groups that underwent immune castration showed normal weight gain within 2 weeks after the initial immunization, indicating the advantage of immunocastration in reducing stress on animals and not affecting their feeding and growth. From the weight gain table (Table 2), it can be observed that the weight gain of the SC group was the lowest among the four groups starting from the 2nd week after immunization. This may be due to the fact that the testosterone level in the SC group is so low that it cannot be detected after castration. Although fighting and climbing behaviors no longer occur, the lack of testosterone also leads to a significant decrease in weight gain. Except for the SC group, the weight gain in the G8 group was significantly lower than that in the NC group. It is speculated that this is also because its testosterone content is significantly lower than that of the NC group, which leads to a slower weight gain. Janett et al [37] used GnRH to actively immunize male lambs and found that the growth rate of immunized castrated lambs was lower than that of the control group. Although the difference was not significant, it was consistent with the results of this experiment. Starting from the 4th week after the first immunization, the weight gain in the LG group was the highest among the four groups. The reason for this may be that, although the testosterone level in the LG group was significantly lower than that in the NC group, it was higher than that in the G8 group. This may be because the serum testosterone level in the LG group was at a suitable level, which can effectively reduce the occurrence of fighting and climbing behavior, reduce energy consumption, and not significantly affect the development of indicators, such as protein, muscle, and bone. The slaughter rate of the SC group is lower than that of the NC group, which may be due to the fact that animals after castration are generally prone to obesity [38,39], and the fat content in the viscera is generally higher, resulting in lower carcass weight despite larger animal weight. After vaccination, there was no significant change in pH in meat, indicating that G8 and LG vaccines did not affect the pH of meat, which is consistent with the results of Gökdal et al [40].

Currently, commercial immunocastration vaccines are mainly used in pigs [23,24] and are not as effective in other species. The development of the two vaccines in this study is expected to fill the gap in immunocastration in the small ruminant neighborhood, and based on the design of the antigenic sequences in this study, these two vaccines are theoretically capable of being used in a wide range of animals, such as swine, cattle, and chickens (the relevant data are still being compiled and analyzed).

In conclusion, these two vaccines are safe and effective vaccines in goats based on the available data. The two immunocastration vaccines developed and prepared in this study effectively inhibited the reproductive ability of male goats, inhibited testicular development and sperm production, and did not affect the growth performance and meat pH of goats. Thus, these are promising and safe immune castration vaccines. The development of these two vaccines is expected to further enrich the theory of immunocastration and to contribute to the promotion of immunocastration to a certain extent.

## Figures and Tables

**Table 1:** Changes in the longitudinal and transverse diameter and circumference of the scrotum in each group of goats

Item (cm)	Group	Initial	4 w	8 w	12 w	16 w	20 w
Growth longitudinal diameter	NC	9.00±0.59	2.15±0.63	2.05±0.47	3.06±0.43^a^	5.16±0.40^Aa^	5.78±0.58^a^
	G8	9.40±0.61	0.65±0.59	0.28±0.63	1.02±0.40^b^	1.02±0.50^B^	1.54±0.87^b^
	LG	8.80±0.34	1.26±0.83	0.86±0.37	1.38±0.73^ab^	2.50±0.74^b^	2.98±0.78^ab^
Growth transverse diameter	NC	6.88±0.23	1.41±0.34	2.06±0.33^A^	2.78±0.18^a^	3.10±0.30^a^	3.45±0.27^a^
	G8	6.62±0.34	0.64±0.19	0.47±0.36^B^	1.04±0.53^b^	1.38±0.69^b^	1.51±0.64^b^
	LG	6.75±0.40	1.28±0.43	1.46±0.47^AB^	1.52±0.58^ab^	1.80±0.29^ab^	2.40±0.29^ab^
Growth circumference	NC	17.60±0.83	3.26±0.59	4.83±0.61^Aa^	6.11±0.79^a^	7.25±0.61^Aa^	7.50±0.66^A^
	G8	17.82±1.09	1.74±0.64	1.00±0.71^B^	2.18±1.15^b^	2.57±1.05^B^	3.21±1.34^B^
	LG	17.16±1.10	1.48±1.01	1.90±1.05^b^	2.30±1.31^b^	2.48±0.55^b^	3.48±0.63^AB^

a,b Different lowercase letters represent p<0.05.

A,B Different uppercase letters represent p<0.01.

NC, non-castration; G8, GnRH8-DTT immunocastration; LG, LHR-GnRH8-DTT immunocastration; GnRH8, GnRH octamer; GnRH, gonadotropin-releasing hormone; DTT, diphtheria toxin T cell helper sequence; LHR, luteinizing hormone receptor.

**Table 2:** Weight gain in different periods of each group of goats

Group	Initial weight (kg)	Net weight gain (kg)						

		1 w	2 w	4 w	8 w	12 w	16 w	20 w
NC	17.60±0.55	1.08±0.20^a^	2.19±0.69^ab^	4.58±1.44	8.54±1.80	13.30±2.10	18.80±2.51	24.25±2.68
G8	17.90±0.77	1.22±0.45^A^	1.68±0.68^ab^	3.20±1.15	7.73±1.48	12.50±1.58	16.79±1.59	21.53±1.60
LG	17.28±0.53	1.31±0.28^A^	2.41±0.46^a^	5.96±0.73	9.99±0.59	15.20±0.87	19.53±0.60	23.32±1.10
SC	16.90±0.77	0.26±0.28^Bb^	0.75±0.41^b^	3.91±0.67	7.50±0.91	11.76±1.12	16.82±1.49	20.76±2.21

a,b Different lowercase letters represent p<0.05.

A,B Different uppercase letters represent p<0.01.

NC, non-castration; G8, GnRH8-DTT immunocastration; LG, LHR-GnRH8-DTT immunocastration; SC, surgical castration; GnRH8, GnRH octamer; GnRH, gonadotropin-releasing hormone; DTT, diphtheria toxin T cell helper sequence; LHR, luteinizing hormone receptor.

**Table 3:** Body sizes of male goats

Item (cm)	Group	Initial	4 w	8 w	12 w	16 w	20 w
Increase in body height	NC	52.80±0.94	2.06±1.32	3.71±1.57	4.66±0.95^b^	10.00±1.54	10.00±2.06^b^
	G8	52.83±1.35	3.18±1.11	4.07±1.22	8.57±1.08^a^	11.12±1.47	12.12±1.06^ab^
	LG	54.00±0.36	3.50±0.85	3.83±1.46	6.38±1.54^ab^	9.13±0.75	14.26±1.10^a^
	SC	53.31±0.82	3.05±1.30	5.26±0.69	7.10±0.70^ab^	9.07±1.01	10.47±1.08^ab^
Increase in body length	NC	53.34±0.67	1.53±1.20	7.30±0.70	6.63±1.75	10.21±1.19	15.06±1.02
	G8	51.97±1.12	1.71±0.57	5.17±1.30	7.92±1.27	10.51±1.66	12.67±1.84
	LG	52.78±1.01	3.64±0.65	6.22±0.83	8.12±1.17	12.42±2.21	15.02±1.39
	SC	52.36±0.90	2.86±1.46	6.30±0.94	6.91±1.43	8.24±0.97	14.35±1.03
Increase in chest circumference	NC	60.95±1.03	6.43±1.73	12.48±2.28	16.55±1.20^a^	20.63±0.90^a^	23.13±1.77
	G8	60.80±1.52	4.45±1.11	8.59±1.23	11.41±1.18^b^	16.85±1.15^ab^	19.74±1.72
	LG	59.28±0.75	6.18±0.85	9.56±1.66	12.50±1.75^ab^	15.06±1.64^b^	18.53±0.92
	SC	60.08±1.45	6.73±0.74	10.34±1.07	12.46±1.25^b^	17.41±2.03^ab^	19.10±1.86
Increase in tube circumference	NC	7.80±0.15	0.40±0.24	0.76±0.24	1.20±0.24	1.65±0.25	2.13±0.24
	G8	7.50±0.15	0.51±0.15	0.57±0.11	1.07±0.15	1.44±0.19	1.68±0.19
	LG	7.35±0.23	0.35±0.15	0.90±0.13	1.50±0.11	1.73±0.13	2.10±0.10
	SC	7.37±0.17	0.48±0.18	0.70±0.19	1.06±0.17	1.36±0.16	1.86±0.18

a,b Different lowercase letters represent p<0.05.

NC, non-castration; G8, GnRH8-DTT immunocastration; LG, LHR-GnRH8-DTT immunocastration; SC, surgical castration; GnRH8, GnRH octamer; GnRH, gonadotropin-releasing hormone; DTT, diphtheria toxin T cell helper sequence; LHR, luteinizing hormone receptor.

**Table 4:** Effect of vaccine on slaughter rate of male goats

Group	Fasting live (weight/kg)	Carcass (weight/kg)	Slaughter rate (%)
NC	41.85±2.88	21.31±1.51	50.91±3.13
G8	40.80±2.30	20.38±1.33	49.80±00.71
LG	42.45±1.36	20.92±0.53	49.38±0.60
SC	38.36±1.78	19.03±0.86	49.67±0.64

Slaughtering rate (%) = carcass weight/fasting live weight×100%.

NC, non-castration; G8, GnRH8-DTT immunocastration; LG, LHR-GnRH8-DTT immunocastration; SC, surgical castration; GnRH8, GnRH octamer; GnRH, gonadotropin-releasing hormone; DTT, diphtheria toxin T cell helper sequence; LHR, luteinizing hormone receptor.

## SUPPLEMENTARY MATERIAL

**Supplement 1**. GnRH8 and LHR sequences.

**Supplement 2**. Plasmid map.

**Supplement 3**. SDS-PAGE electropherogram of protein induced purification.
