# Supplementary material for: Preparation of two kinds of immunocastration vaccines and their immune effects on male goats
Source: Anim Biosci. 2025 Apr 11;38(7):1411–21. doi: 10.5713/ab.24.0811 (PMC12229912; doi:10.5713/ab.24.0811)
Supplement: Supplementary file 1 [file ab-24-0811-Supplementary-1.pdf]

A

GAACATTGGTCTTATGGTCTGCGTCCAGGTGGTGGTTCTGAAC
ACTGGTCTTATGGTCTGCGTCCTGGTGGTGGTTCTCAGCATTG
GAGCTATGGTCTGCGCCCAGGCGGTGGTAGCTACCATTGGTC
TCACGGTCTGCGTCCGGGTGGTGGTAGCGAACATTTCTCCTA
TTCCCTGGTTCCGGGTGGCGGTTCCCAGCATTGGTCCTATGGC
CTGCGTCCGGCTGGTGGTTCTGAACACTGGTCTTATGGTTGGC
GCCCGGCTGGCGGTTCTGAACATTGGTCTTACGGTGTACGCC
CGGCTGGTGGTGGTGGTTCT

B

ATGGAAATCTCCCAATCTGACTCTCTGGAAAAGATCGGTGG
TTCTAGCTATTCCCTGAAAAAACTGCCGTCTCGCGGTGGTG
GTGGTTCTGAAATCTCCCAATCTGACTCTCTGGAAAAGATC
GGTGGTTCTAGCTATTCCCTGAAAAAACTGCCGTCTCGCGG
TGGTGGTGGTTCTGAAATCTCCCAATCTGACTCTCTGGAAA
AGATCGGTGGTTCTAGCTATTCCCTGAAAAAACTGCCGTCT
CGCGGTGGTGGTGGTTCT

C

Ehwsyglrpgggsehwsyglrpgggsqhwsyglrpgggsyhws
hglrpgggsehfsyslvpgggsqhwsyglrpaggsehwsygwr
paggsehwsygvrpaggggs

D

meisqsdslekiggssyslkklpsrggggseisqsdslekiggs
syslkklpsrggggseisqsdslekiggssyslkklpsrggggs

E

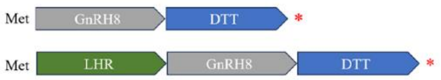

Supplement 1. GnRH8 and LHR sequences. A. GnRH8 Nucleic Acid Sequence; B. LHR Nucleic Acid Sequence; C. GnRH8 Amino Acid Sequence; D. LHR Amino Acid Sequence; E. Schematic diagram of the structure of GnRH8-DTT and LHR-GnRH8-DTT.
