# Supplementary figures and images for: Preparation of two kinds of immunocastration vaccines and their immune effects on male goats
Source: Anim Biosci. 2025 Apr 11;38(7):1411–21. doi: 10.5713/ab.24.0811 (PMC12229912; doi:10.5713/ab.24.0811)

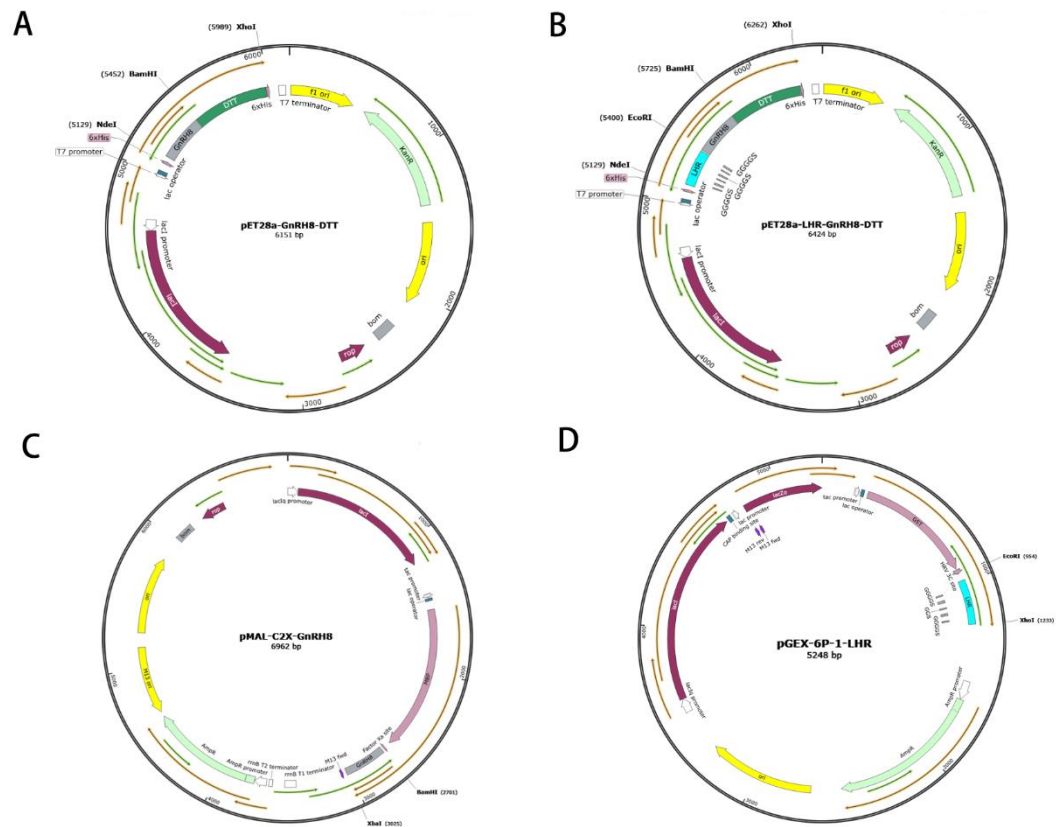

Supplement 2. Plasmid map. A. pET28a-GnRH8-DDT; B. pET28a-LHR-GnRH8-DDT; C. pMAL-C2X-GnRH8; D. pGEX-6P-1-LHR

Supplement: Supplementary file 2 [file ab-24-0811-Supplementary-2.pdf]
