# Supplementary material for: Preparation of two kinds of immunocastration vaccines and their immune effects on male goats
Source: Anim Biosci. 2025 Apr 11;38(7):1411–21. doi: 10.5713/ab.24.0811 (PMC12229912; doi:10.5713/ab.24.0811)
Supplement: Supplementary file 3 [file ab-24-0811-Supplementary-3.pdf]

The constructed recombinant plasmids were transformed into BL21 receptor cells, and after induced expression and purification, SDS-PAGE electrophoresis was performed. As shown in Supplement 3, the four plasmids constructed were transformed and induced by IPTG, all of them had obvious induced bands at the target size, and after purification, the corresponding size of the protein was obtained.

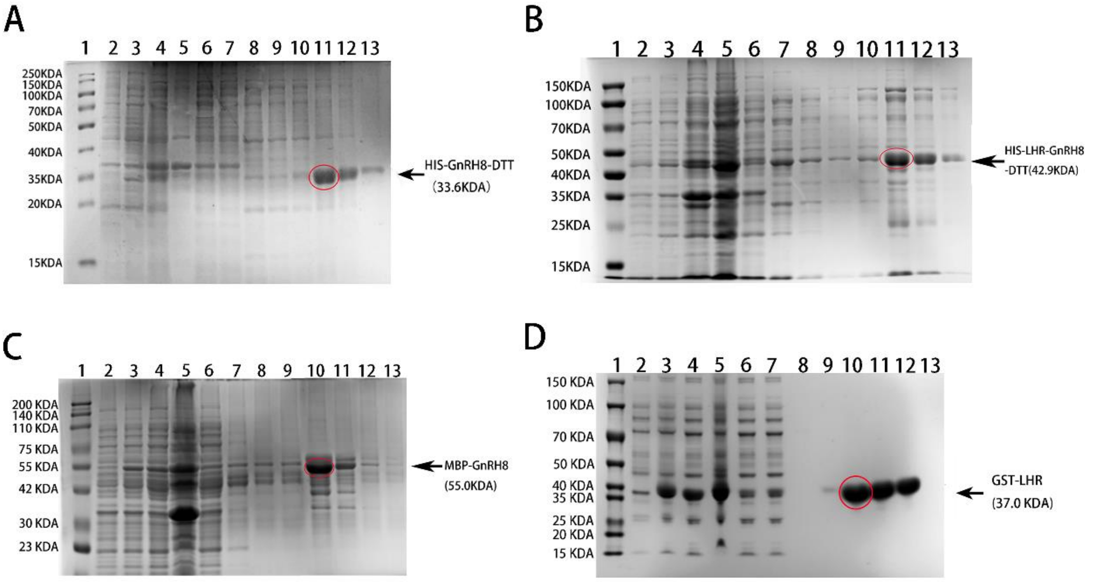

Supplement 3. SDS-PAGE electropherogram of protein induced purification

Figure Note: A: HIS-GnRH8-DTT protein purification； B: HIS-LHR-GnRH8-DTT protein purification; C: MBP-GnRH8 Protein purification；D: GST-LHR protein purification. 1 canal: marker; 2 canal: pre-induction bacterial fluid; 3 canal: post-induction bacterial fluid; 4 canal: supernatant after fragmentation of induced bacterial bodies; 5 canal: precipitate after fragmentation of induced bacterial bodies; 6 canal: fluidized Kawasaki fluid remaining after hanging the column with the protein solution; 7 canal: first eluent; 8 canal: second eluent; 9 canal: third eluent; 10 canal: first protein eluent; 11canal: second protein eluent; 12 canal: third protein eluent; 13 canal: fourth protein eluent; Red circles: corresponding target proteins.
